# Pure Isolated Internuclear Ophthalmoplegia as Presentation of Midbrain Ischemic Stroke: A Case Report

**DOI:** 10.7759/cureus.47083

**Published:** 2023-10-15

**Authors:** Muzamil Musa, Leena Saeed, Sondos K Khalil, Nawras Hatam Mahdy Al-tikrety, Zahra B Yousif, Yasmin Ahmed, Motaz M Almahmood, Amjad Salman

**Affiliations:** 1 Internal Medicine, Hamad Medical Corporation, Doha, QAT; 2 Internal Medicine, Hamad General Hospital, Doha, QAT; 3 Internal Medicine, Qatar University, Doha, QAT; 4 Internal Medicine, Royal Care International Hospital, Khartoum, SDN

**Keywords:** ophthalmoplegia, midbrain infarction, ischemic stroke, ino, internuclear ophthalmoplegia

## Abstract

Internuclear ophthalmoplegia (INO) is a condition characterized by impaired ocular movement, leading to an inability to perform coordinated lateral gaze, resulting in ophthalmoplegia. This impairment results from damage to the medial longitudinal fasciculus (MLF), which can occur because of various types of lesions localized in the pons or midbrain. In this case, we report on a 67-year-old man with multiple comorbidities who arrived at the emergency department with complaints of sudden dizziness and an unsteady gait. During the examination, he exhibited left INO, which was characterized by limited left eye adduction and multidirectional nystagmus of the right eye when performing right lateral gaze.

## Introduction

The human brain is intricately mapped, with every location having its own function. Internuclear ophthalmoplegia (INO) is a condition characterized by impaired ocular movement, resulting in the inability to execute coordinated lateral gaze and ophthalmoplegia. This impairment arises from damage to the medial longitudinal fasciculus (MLF). INO stemming from a midbrain infarction is a rare clinical presentation. Adduction may be preserved during convergence. Skew deviation or hypertropia on the side of the lesion is often present [1-2]. A lesion involving the medial longitudinal fasciculus (MLF) at the brainstem, which consists of crossed-over tracts connected with ocular nuclei, is implicated as the cause of INO. INO can have multiple causes, such as multiple sclerosis (MS), tumors, infections, hydrocephalus, trauma, nutritional or metabolic disorders, and vascular diseases, primarily localized in the pons or midbrain [2-3]. However, isolated INO from infarction is not frequently reported in the literature. Hence, the significance of our case report becomes evident.

## Case presentation

A 67-year-old male with past medical history of hypertension, diabetes, and dyslipidemia presented to the emergency department complaining of an acute onset of dizziness for approximately six hours, associated with an unsteady gait. This presentation was not associated with head movements, headaches, nausea, vomiting, or visual problems. He denied any loss of consciousness, speech, motor, or sensory symptoms. There was no ear pain or tinnitus, but there was chronic gradual hearing loss mainly attributed to aging. He denied any cough, chest pain, shortness of breath, palpitations, abdominal pain, or changes in bowel habits.

On examination, his vital signs were stable. He was afebrile with a temperature of 36.8 degrees Celsius, had a pulse rate of 73 beats per minute, a respiratory rate of 18 breaths per minute, and a blood pressure of 131/61 mmHg. He remained conscious, alert, and fully oriented to time, place, and person. His Glasgow coma score was 15/15. Pupillary responses were normal and equal, reacting appropriately to light stimulation. During the examination of his eye movements, we noted specific findings. In the right gaze, there was limited adduction of the left eye, along with multidirectional nystagmus in the right eye, consistent with left INO. However, during the left gaze, the right eye showed normal adduction, and no nystagmus was observed in the left eye. Additionally, comprehensive neurological assessments, including evaluations of cognitive function, motor skills, sensory perception, coordination, and gait, were normal.

The electrocardiogram was normal except for left ventricular hypertrophy. An echocardiography revealed an ejection fraction of 55%. A computed tomographic angiogram (CTA) of the head was performed, revealing neither acute lesion nor evidence of significant stenosis or occlusion. The patient's laboratory testing showed a normal complete blood count, renal function test, and electrolytes. His hemoglobin A1C was elevated (8.7%), and he had a normal lipid profile as seen in Table 1.

**Table 1:** Laboratory blood test results of the patient HbA1C: hemoglobin A1C; LDL: low-density lipoprotein; HDL: high-density lipoprotein

Test	Results	Lab reference
White Blood Cells	8.8 x10^3/uL	4.0-10.0 x10^3/uL
Hemoglobin	14.3 gm/dL	13.0-17.0 gm/dL
Platelets	280 x10^3/uL	150-410 x10^3/uL
Urea	5.9 mmol/L	2.5-7.8 mmol/L
Creatinine	92 umol/L	65 -105 umol/L
Sodium	138 mmol/L	135-145 mmol/L
Potassium	3.8 mmol/L	3.5-5.0 mmol/L
Adjusted Calcium	2.40 mmol/L	2.10-2.60 mmol/L
HbA1C%	8.7%	<5.7%
Cholesterol	4.8 mmol/L	< 5.2 mmol/L
LDL	2.4 mmol/L	< 2.59 mmol/L
HDL	1.3 mmol/L	>1.0 mmol/L

**Figure 1:**
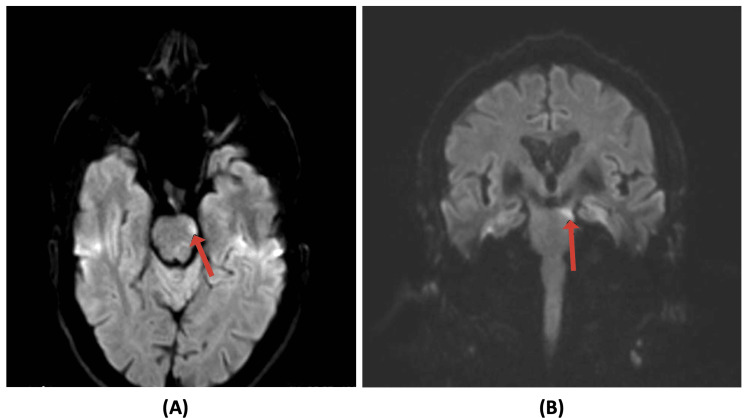
Diffusion-weighted images demonstrating a diffusion restriction (indicated by red arrows) at the left anterior aspect of the midbrain and left medial longitudinal fasciculus seen in axial (A) and coronal (B) planes.

**Figure 2:**
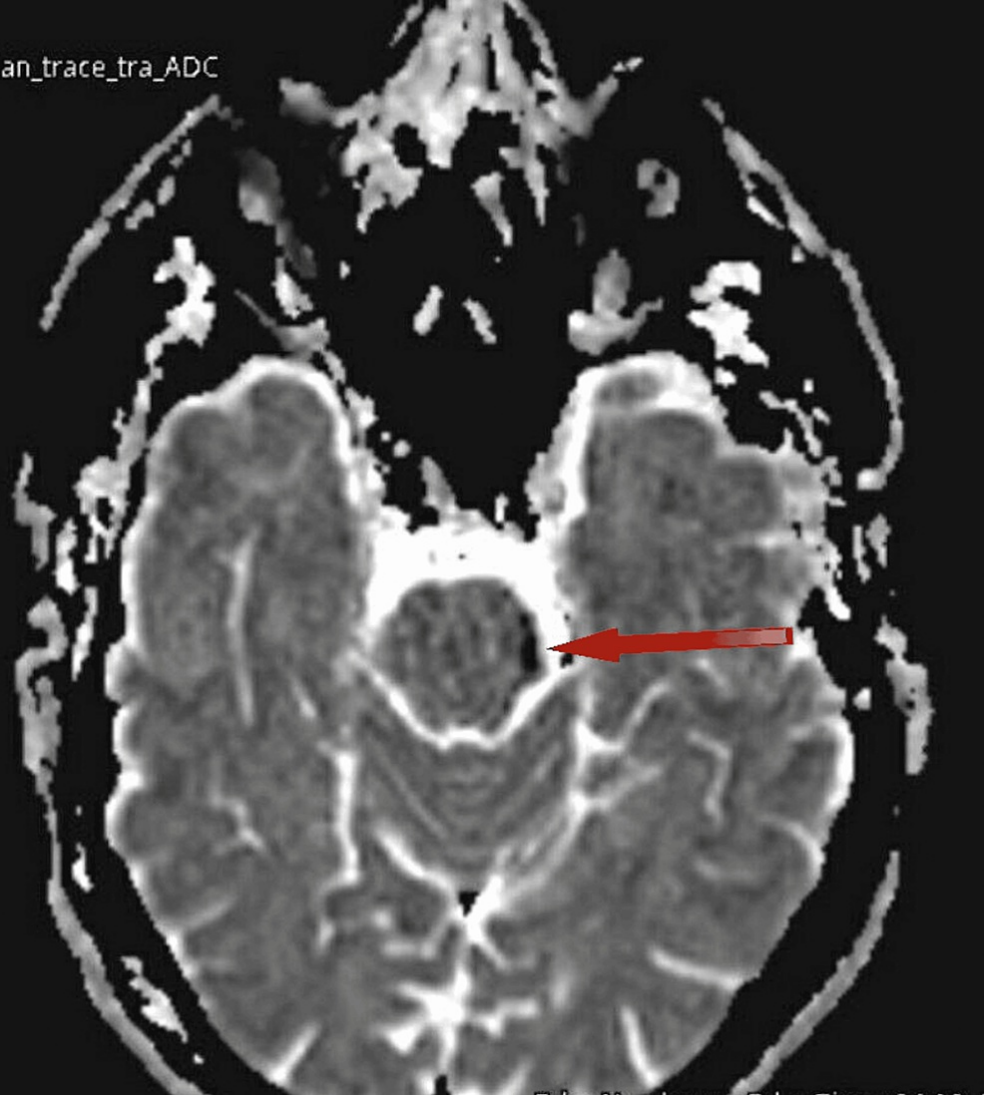
An ADC MRI image demonstrating low signal indicating ischemic infarct (red arrow) ADC: apparent diffusion coefficient; MRI: magnetic resonance imaging

An MRI of the brain showed a small focal area of diffusion restriction with mild DWI bright and ADC map dark signals at the left lower anterior aspect of the midbrain, which suggested early acute infarct (Figures 1-2).

The stroke was managed with aspirin 100 mg and atorvastatin 40 mg once daily, in addition to minor modifications to the antidiabetic regimen. His hospital stay was uneventful, and his dizziness improved. After two days of admission, he was followed by an occupational therapist and transferred to a rehabilitation center.

## Discussion

Our case describes a 67-year-old male with multiple comorbidities who presented with dizziness and was then found to have unilateral internuclear ophthalmoplegia (INO) secondary to an isolated midbrain infarction. Overall, mesencephalic infarction is a rare site of stroke in comparison to other parts of the brain because of the intricate arterial blood supply to the mesencephalon.

The midbrain receives its blood supply from various branches originating from the posterior cerebral artery (PCA), upper basilar artery (BA), and superior cerebellar artery. In cases of ischemic stroke in the posterior circulation, the midbrain is frequently affected. Usually, it is accompanied by the involvement of neighboring structures such as the pons, thalamus, and cerebellum [4]. However, isolated midbrain infarctions are rare [5-6].

Kim et al. performed a study on 40 patients with infarcts limited to the midbrain and found that gait ataxia is the most common clinical manifestation of midbrain strokes (68%), followed by dysarthria (55%), limb ataxia (50%), sensory symptoms (43%), third nerve palsy (35%), and limb weakness (23%). Only 13% of patients in the study reported INO [7].

The midbrain is divided into three zones: paramedian, lateral, and posterior zones, based on the mesencephalon blood supply. In a case study conducted by Zhang et al., 25 patients with isolated INO resulting from a pure midbrain infarction were examined. The study found that the ratio of adduction palsy was significantly higher in paramedian midbrain infarction (62.5%) compared to non-paramedian midbrain infarction (11.8%) [8]. These findings were also observed in a study by Kim et al., where five cases of internuclear ophthalmoplegia were caused by the involvement of the medial longitudinal fasciculus (MLF) in the paramedian and lower midbrain areas [7]. Our patient had a left lower anterior midbrain infarction in the lateral zone.

The predominant etiology of INO is attributed to small acute infarcts primarily located in the posterior region of the pons. This localization aligns with the anticipated position of the brain-stem pathways [9]. At the midbrain level, INO occurs when the MLF is affected, which is located in the paramedian zone. However, the exact pathophysiology of INO due to lateral midbrain infarction is unclear. This can be elucidated by accompanying lesions at a level responsible for INO secondary to the non-horizontal direction of the penetrating vessels [10] or due to the involvement of proximal pathways that control eye movement, which may be limited by the narrow space between midbrain structures.

Approximately one-third of INO cases result from demyelinating disorders such as multiple sclerosis, which predominantly affect both sides and are commonly seen in young adults. Another one-third of cases are attributed to infarctions, typically affecting only one side and more commonly observed in older individuals [11].

In addition to the finding of horizontal gaze palsy in the physical examination, the diagnosis of our patient was supported by his age and medical history of hypertension, diabetes mellitus (DM), dyslipidemia, and a recurrent history of strokes. Diabetic patients were found to have approximately 2.6 times higher odds of experiencing brainstem infarction compared to non-diabetic patients. Specifically, mono-focal brainstem infarctions were observed to be 3.3 times more prevalent in DM patients when compared to non-DM patients [12]. Our patient has a 20-year history of uncontrolled DM, which contributes to atherogenesis and autonomic neuropathy [13].

## Conclusions

In conclusion, INO is a rare clinical presentation of isolated midbrain infarction. To accurately diagnose the underlying cause of INO and detect incipient strokes, it is crucial to follow a systematic clinical approach, which involves obtaining a comprehensive medical history and conducting a thorough physical examination. Furthermore, understanding the relationship between associated clinical neurological findings and the underlying anatomy is essential for an accurate diagnosis.

## References

[1] Smith JW, Cogan DG (1959). Internuclear ophthalmoplegia; a review of fifty-eight cases. AMA Arch Ophthalmol.

[2] Leigh RJ, Zee DS (497-511). The Neurology of Eye Movements. Press.

[3] Bassetti C, Bogousslavsky J, Barth A, Regli F (1996). Isolated infarcts of the pons. Neurology.

[4] Caplan LR (1980). "Top of the basilar" syndrome. Neurology.

[5] Bogousslavsky J, Maeder P, Regli F, Meuli R (1994). Pure midbrain infarction: clinical syndromes, MRI, and etiologic patterns. Neurology.

[6] Martin PJ, Chang HM, Wityk R, Caplan LR (1998). Midbrain infarction: associations and aetiologies in the New England Medical Center Posterior Circulation Registry. J Neurol Neurosurg Psychiatry.

[7] Kim JS, Kim J (2005). Pure midbrain infarction: clinical, radiologic, and pathophysiologic findings. Neurology.

[8] Zhang Y, Wang L, He M (2017). Isolated INO as a presentation of midbrain paramedian area lacunar infarction in patients with diabetes. J Clin Neurosci.

[9] Bae YJ, Kim JH, Choi BS, Jung C, Kim E (2013). Brainstem pathways for horizontal eye movement: pathologic correlation with MR imaging. Radiographics.

[10] Kim JS (2004). Internuclear ophthalmoplegia as an isolated or predominant symptom of brainstem infarction. Neurology.

[11] Feroze KB, Wang J (2023). Internuclear Ophthalmoplegia. https://www.ncbi.nlm.nih.gov/books/NBK441970/.

[12] Ichikawa H, Kuriki A, Kinno R (2012). Occurrence and clinicotopographical correlates of brainstem infarction in patients with diabetes mellitus. J Stroke Cerebrovasc Dis.

[13] Parkinson FE, Hatch GM (2016). Is there enhanced risk of cerebral ischemic stroke by sulfonylureas in type 2 diabetes?. Diabetes.

